# Selective Impairment in Frequency Discrimination in a Mouse Model of Tinnitus

**DOI:** 10.1371/journal.pone.0137749

**Published:** 2015-09-09

**Authors:** Laetitia Mwilambwe-Tshilobo, Andrew J. O. Davis, Mark Aizenberg, Maria N. Geffen

**Affiliations:** Department of Otorhinolaryngology HNS, University of Pennsylvania, Philadelphia, Pennsylvania, United States of America; University of Salamanca- Institute for Neuroscience of Castille and Leon and Medical School, SPAIN

## Abstract

Tinnitus is an auditory disorder, which affects millions of Americans, including active duty service members and veterans. It is manifested by a phantom sound that is commonly restricted to a specific frequency range. Because tinnitus is associated with hearing deficits, understanding how tinnitus affects hearing perception is important for guiding therapies to improve the quality of life in this vast group of patients. In a rodent model of tinnitus, prolonged exposure to a tone leads to a selective decrease in gap detection in specific frequency bands. However, whether and how hearing acuity is affected for sounds within and outside those frequency bands is not well understood. We induced tinnitus in mice by prolonged exposure to a loud mid-range tone, and behaviorally assayed whether mice exhibited a change in frequency discrimination acuity for tones embedded within the mid-frequency range and high-frequency range at 1, 4, and 8 weeks post-exposure. A subset of tone-exposed mice exhibited tinnitus-like symptoms, as demonstrated by selective deficits in gap detection, which were restricted to the high frequency range. These mice exhibited impaired frequency discrimination both for tones in the mid-frequency range and high-frequency range. The remaining tone exposed mice, which did not demonstrate behavioral evidence of tinnitus, showed temporary deficits in frequency discrimination for tones in the mid-frequency range, while control mice remained unimpaired. Our findings reveal that the high frequency-specific deficits in gap detection, indicative of tinnitus, are associated with impairments in frequency discrimination at the frequency of the presumed tinnitus.

## Introduction

Tinnitus is an auditory disorder, characterized by a perception of sound in the absence of external acoustic stimulation. Tinnitus affects nearly 50 million adults in the United States [1], with 10–15% suffering from a severe form of the disorder [2]. Whereas the phantom auditory percept varies between individuals, it is typically experienced as a continuous or intermittent ringing or buzzing sound in the ears centered around 3–8 kHz [3]. Chronic tinnitus can be debilitating, leading to disturbances in sleep, impaired concentration [4], and depression [5].

Typically triggered by repeated exposure to loud sounds [1], tinnitus is commonly accompanied by structural and functional neurological changes induced as a result of acoustic trauma and leading to hearing loss [6, 7]. Tinnitus incidence is greatly increased in patients with hearing impairments in the mid-to-high frequency range [1]. It is likely that changes in neuronal response properties in the auditory cortex as a result of acoustic trauma, contribute to the generation of tinnitus. High frequency hearing loss also affects auditory processing, as seen by the enhancement in frequency discrimination in frequency bands bordering the hearing loss region [8]. Neuroimaging of individuals with noise-induced hearing loss reveals reorganization of cortical responses to sounds, possibly indicating changes in the tuning properties of auditory neuronal populations [9, 10]. Measurements of cognitive control in tinnitus patients further point to impaired sound perception. When compared to age matched controls, tinnitus patients demonstrate deficits in executive control [11], processing speed, and reaction time of auditory sensory information [4, 12]. However, little is understood about the mechanisms of how tinnitus impacts auditory perception.

The use of animal models has considerably advanced our current understanding of the neural changes associated with tinnitus. Tinnitus-like symptoms can be induced in rodents following prolonged noise or tone exposure [13, 14]. In the animal models, tinnitus is identified as a selective deficit when detecting a silent gap in a narrow-band noise sequence, specific for high frequencies [15]. The presence of tinnitus is expected to “fill in” the gap in noise. It can be measured by reduced attenuation of the acoustic startle reflex (ASR) due to a gap in narrow-band noise that precedes the startle noise stimulus [13–15]. In mice exhibiting tinnitus, tuning of neurons to the tinnitus frequency is diminished, and is thought to shift outside the tinnitus range [16]. Recently, we demonstrated that learning-evoked changes in frequency discrimination acuity are controlled by the activity of the auditory cortex [17], likely driven by tuning of cortical neurons [18, 19]. We hypothesized that frequency discrimination may also be affected in mice exhibiting tinnitus.

The goal of the present study was to characterize the effect of tone-induced tinnitus on frequency discrimination. Tinnitus was induced in mice through prolonged exposure to a 10 kHz frequency tone and assayed through a gap detection test in mid-range and high frequency bands. The test tones for the two ranges were designed to overlap, spanning 12–16 kHz and 15–22 kHz, respectively. Frequency discrimination was assessed by using a modified behavioral paradigm based on measuring pre-pulse inhibition of ASR [17, 20]. We compared frequency discrimination acuity prior to tone exposure, and at 1, 4 and 8 weeks post tone-exposure. We found that tone-induced tinnitus in mice impaired auditory frequency discrimination in the high-range frequency band, corresponding to tinnitus (in which gap detection was impaired), and mild impairments in the frequency range close to the tone to which the mice were exposed.

## Materials and Methods

### Mice

Adult male CBA/J mice (Jackson Laboratories) of approximately 7–9 weeks of age were housed in a temperature-controlled (26°C) vivarium maintained at a 12 h light/dark cycle, with ad *libitum* access to food and water. All experimental procedures were conducted during the mice's dark cycle. All procedures were approved by the Institutional Animal Care and Use Committee at the University of Pennsylvania.

### Experimental Design

**Fig 1:**
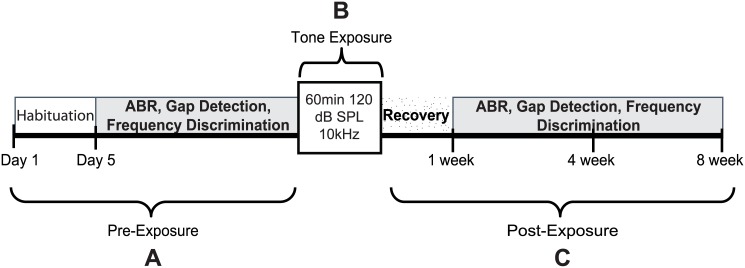
Timeline of the experimental protocol and testing. (A) Habituation to the test environment and apparatus, baseline recording of auditory brainstem responses (ABRs), and behavioral testing for gap detection and frequency discrimination; (B) 60 min tone exposure to a 10kHz tone; (C) Post-exposure ABR recording, gap detection, and frequency discrimination testing at 1 week, 4 weeks, and 8 weeks.

Mice were first habituated to the testing environment and apparatus over a four-day period (Fig 1). Measurement of auditory brainstem response (ABR) thresholds to tone pips was followed by behavioral testing of gap detection and frequency discrimination of mid-range and high-range frequency tones (FD-medium; FD-high). Baseline behavioral responses for each mouse were computed and averaged over three sessions. Once baseline testing was completed, mice were separated into two groups, Control (N = 6) and Exposed (N = 22). Tinnitus was induced in mice during a one-hour exposure to a loud continuous tone. ABRs, gap detection, and frequency discrimination was conducted at three different time points post-exposure: 1, 4, and 8 weeks. Previous studies have shown that tone and noise exposure does not induce tinnitus in all mice [13, 14]. Therefore, the exposed mice were further divided into a Tinnitus(+) group if behavioral signs of tinnitus were observed at 4 weeks post-exposure (N = 14), and Tinnitus(-) group if no behavioral signs of tinnitus were present at 4 weeks post-exposure (N = 8).

### Habituation

On the first day of habituation, a custom-made transparent polycarbonate slotted restrainer was placed within each home cage. On the second day, each mouse was individually placed within the restrainer for 25 minutes inside the sound isolation booth. Auditory stimuli were not presented on the second day; however, on days 3 and 4 of habituation a 60 dB SPL white noise was presented throughout the 25 minute habituation period from a speaker positioned approximately 10 cm above the restrainer. Baseline gap detection and frequency discrimination were conducted over a three week period.

### Apparatus

Behavioral testing was performed inside a sound isolated chamber. All auditory stimuli used for behavioral testing were generated by MatLab software and calibrated with a Bruёl & Kjӕr ¼ inch free-field microphone (Type 4939). Auditory stimuli were delivered with a MF1 Multi Field Magnetic speaker (TDT Systems) powered by a three-watt stereo amplifier (SA1 Stereo Amplifier, TDT Systems). Each mouse was placed in a clear polycarbonate restrainer mounted to an acrylic base. The restrainer was placed on top of the startle platform (San Diego Instruments, CAL005371). The speaker was positioned approximately 10 cm above the restrainer. An output voltage of the force of the mouse startle response was recorded by a transducer mounted on the underside of the platform. Data from the startle platform was continuously recorded throughout each session and down-sampled by 100x. The mouse ASR was recorded and analyzed by MatLab as auditory stimuli were generated. All mice were monitored during behavioral testing via a webcam (Logitech Quick Cam Pro).

### Tone Exposure

Tinnitus was induced during a 1-hour exposure session to a 10 kHz pure tone delivered at 120 dB sound pressure level relative to 20 micro Pa (SPL). Since tone exposure can have detrimental effects on hearing [14, 21], the right ear of each mouse was plugged with a silicon ear plug prior to tone exposure. Mice in the exposed group were awake and freely moving throughout the exposure session. Each mouse was individually placed within the exposure chamber. The tone was delivered through a Pyramid TW67 Speaker powered by a Crown XLS 802 power amplifier, positioned directly against the exposure chamber. Once tone exposure was complete, mice were allowed to recover for 45 min and were then returned to their home cage.

### Gap Detection

Gap detection was used to assay tinnitus-like symptoms in mice. Each session began with a 5-minute acclimation period during which a 60 dB SPL broadband background noise was played continuously. Each session consisted of 98 trials. In order to ensure that the mouse’s ASR was not significantly different at the start and end of the session, the first and the last trials were Startle-Only (SA) trials. The remaining 96 trials were experimental trials. They consisted of 8 SA trials and 8 Gap (GA) trials for each of 6 frequencies (6–8 kHz, 10–12 kHz, 14–16 kHz, 18–20 kHz, 26–28 kHz, and 2–32 kHz broadband noise (BBN); 60 dB SPL), randomly intermixed. On SA trials, a continuous sound was presented at one of the frequency bands for 10–20 s prior to a 115 db SPL startle noise burst that was 50 ms long. Background noise of the same frequency band then resumed immediately after the end of the startle stimulus (SS) for 500 ms. The GA trials were similar to the SA trials except that a 40 ms silent gap was presented 100 ms prior to the onset of the SS. Mice were allowed a 24–48 hour rest period between sessions during any given data collection period.

The amplitude of the ASR was defined as the maximum vertical force applied during 550 ms following SS onset minus the average activity during the 500 ms preceding SS. The average ASR amplitude for all GA and SA trials and their respective standard error of the mean were calculated for each of the six frequency bands within a given session. Percent inhibition was computed as:

$$\text{ASRinhibition}\left(\text{\%}\right)=100\times \frac{SA-GA}{SA}$$

In order to account for excessive movement throughout a session, the standard deviation (SD) of the startle data for each trial was analyzed for the 9 s preceding the onset of the SS. If the SD for a given trial exceeded the average value for all trials of the same frequency band during a given session by more than 30%, the trial was eliminated and not considered in further calculations.

The %ASR-inhibition values for each mouse was averaged across all sessions for each frequency band. Individual compilations for each group were pooled into population data within each frequency band. Any significant decrease in %ASR-inhibition from baseline indicates deficits in gap detection in that frequency band, suggesting the presence of tinnitus in that frequency band.

### Auditory Brainstem Response

ABR thresholds to tone pips were used to measure hearing after unilateral tone exposure. Mice were anesthetized with isoflurane (1–2.5%) and electrodes were inserted subdermally (recording electrode—posterior to the pinna of the exposed ear; reference electrode—anterior cranial midline; ground electrode—posterior cranial midline). Thresholds were obtained for tone pips at 8, 12, 16, 22, 28 kHz (5 ms in duration, 0.1 ms ramp; 1200 repetitions) presented from 10–80 dB SPL in 10dB steps. ABR signals were amplified, filtered (band passed 300 Hz-2000 Hz), and averaged using Neuralynx (Cheetah software).

### Frequency Discrimination

Discriminability of mid-range and high frequency tones was assessed using an ASR-based protocol [17, 20]. Mice were tested on two separate frequency discrimination tasks: FD-medium and FD-high. Both tasks followed a similar protocol but differed in the background and test frequencies used. In the FD-medium sessions the background frequency was a 12 kHz 70 dB background tone with the following pre-pulse frequencies: 15.84 kHz, 13.84 kHz, 13.20 kHz, 12.96 kHz, 12.72 kHz, 12.48 kHz, 12.24 kHz, 12.12 kHz, and 12 kHz. In the FD-high sessions, the background frequency was a 22000 kHz 70 dB background tone with the following pre-pulse frequencies: 14.96 kHz, 18.48 kHz, 19.80 kHz, 20.24 kHz, 20.68 kHz, 21.12 kHz, 21.56 kHz, 21.78 kHz, and 22 kHz. Each test session started with 9 SA trials, in which a 70 dB background tone was followed by a 120 dB, 20 ms-long broadband startle stimulus. The SA trials were followed by 120 pre-pulse trials presented in a pseudorandom order with an inter-trial interval varying randomly between 10 and 20 s. On pre-pulse trials, the pre-pulse stimulus was one of the six pre-pulse frequencies presented for 80 ms before SS onset. A 1 ms ramp between the background tone and the pre-pulse tone was included in order to avoid clicks. The test session was concluded with an additional SA trial.

Pre-pulse inhibition (PPI) for each frequency was measured based on the amplitude of the mouse’s ASR. The ASR was measured similarly as for the gap detection test. For each PPI session, the ASRs for each frequency were averaged and used to calculate the percent of inhibition:

$$\text{PPI}\left(\text{\%}\right)=100\times \frac{ASRnopps-ASRpps}{ASRnopps}$$

where ASRnopps is the response when pre-pulse frequency is equal to the frequency of the background tone and ASRpps is the response after a frequency shift has occurred.

The frequency discrimination threshold (Th) was defined as a frequency shift that caused 40% inhibition of the maximum ASR. Th is determined from a parametric fit to a generalized logistic function:

$$\text{PPI}=\;-\frac{a}{2}+\frac{a}{1+\text{exp}\left(b+c\Delta f\right)}$$

The frequency shift is expressed as the percent change in frequency relative to the background tone (Δ*f*,%):

$$\Delta f,\%=100\times \;\frac{\left|background\;frequency-test\;frequency\right|}{background\;frequency}$$

### Data Analysis

To identify mice that developed tinnitus following tone exposure, startle responses on GA trials were compared with their corresponding SA responses at 4 weeks post-exposure using a paired t-test. Exposed mice that demonstrated a significant decrease in gap detection (data pulled over all frequencies measured, paired t-test, significance at *p*<0.05) were placed in the Tinnitus(+) group, and those that did not demonstrate a significant decrease in gap detection were placed in the Tinnitus(-) group.

In order to determine the effects of tone-induced tinnitus on gap detection, the data was analyzed using a two-way repeated measures analysis of variance with time (baseline, 1 week post-exposure, 4 weeks post-exposure, and 8 weeks post-exposure) and frequency (2–32 kHz, 6–8 kHz, 10–12 kHz, 14–16 kHz, 18–20 kHz, 26–28 kHz) as factors. Significant main effects and interactions were further analyzed by paired sample t-test and adjusted using Bonferroni correction. Samples that did not pass the Mauchly’s test for sphericity were compared using Greenhouse-Geisser correction. To distinguish between tinnitus and hearing loss, ABR thresholds in the exposed ear of a subset of Tinnitus(+) and Tinnitus(-) mice were assayed using a paired t-test.

The effect of tone-induced tinnitus on discriminability of mid-range and high-range frequency tones were assessed for each group using a repeated measure ANOVA with timepoint (baseline, 1 week post-exposure, 4 weeks post-exposure, and 8 weeks post-exposure) as the within-subject factor. Significant changes in post-exposure frequency discrimination were further analyzed by paired t-test in order to identify frequency specific differences in PPI. All statistical analyses were done using SPSS Statistics (IBM) or Matlab. Effects were considered statistically significant at p≤ 0.05 unless otherwise stated.

The frequency discrimination Th for each post-exposure time was compared to baseline by using a parametric bootstrap estimate on the basis of 1000 draws of the data from the mean and standard deviation of the measured ASR at different frequencies. Significance was considered if the mean of the measurement was at least one standard deviation away from the mean at baseline.

## Results

### Gap detection impairments following tinnitus induction

To induce tinnitus, we exposed mice to a continuous prolonged loud tone (10 kHz 120 dB SPL, 1 hr). Detection of a gap in noise, measured as inhibition of auditory startle response, was used to determine tinnitus-like behavior in mice. Normally, a gap in a continuous background noise that precedes a startle noise inhibits ASR. If the animal experiences a phantom noise at a specific frequency, the animal is expected to exhibit poorer detection of the silent gap embedded in noise at that frequency and therefore decreased inhibition of ASR relative to SA trials. In previous studies, gap detection was impaired within specific frequency bands following tone exposure in a subset of animals and interpreted as behavioral evidence of tinnitus [13, 14, 22, 23]. We tested gap detection in narrow noise bands of 6–8, 10–12, 14–16, 18–20 and 26–28 kHz, as well as in broadband noise between 2–32 kHz, at baseline (prior to exposure), 1 week, 4 weeks and 8 week post-exposure. We used the measurements at 4 weeks, which is the time when chronic tinnitus is expected to develop [24], to assign exposed mice in Tinnitus(+) and Tinnitus(-) groups. Mice in a Control group (N = 6) underwent the same testing, but were placed in a chamber with no auditory stimulus presentation instead of continuous tone exposure.

**Fig 2:**
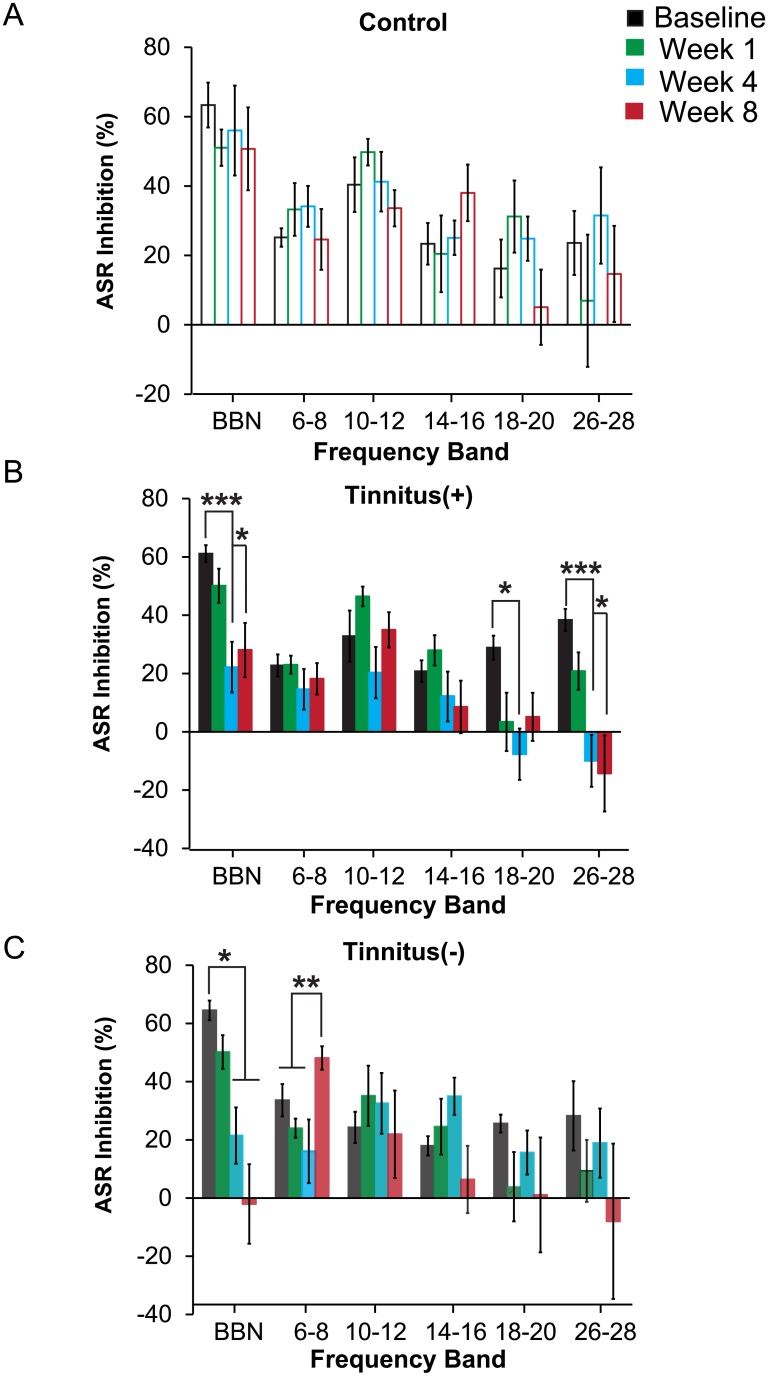
Gap detection of the pre-pulse induced inhibition of the acoustic startle response (ASR) for Control (N = 6), Tinnitus(+) (N = 14), and Tinnitus(-) (N = 8) mice at baseline, and 1, 4, and 8 weeks post-exposure. (A) Control mice exhibited no significant changes within each test frequency when comparing baseline and post-exposure ASR-inhibition. (B) Tinnitus(+) mice exhibited evidence of decreased performance on gap detection in high frequency bands after tone exposure. ASR-inhibition was significantly attenuated at 4 weeks post-exposure for the 2–32 kHz (BBN), 18–20 kHz, and 26–28 kHz bands, and at 8 weeks post-exposure for the 2–32 kHz and 26–28 kHz bands. (C) Tone exposure did not attenuate gap detection at high frequencies in Tinnitus(-) mice. Instead, Tinnitus(-) mice demonstrated gap detection deficits for BBN at 4 and 8 weeks post-exposure, and improved detection in the 6–8 kHz band at 8 weeks post-exposure. Each data point represents population mean ± SEM. *: p<0.05; **: p<.01.

Out of the tone-exposed mice (N = 22), 14 mice developed a significant decrease in gap detection (*p*< 0.001) at 4 weeks post-exposure and were placed in the Tinnitus(+) group (Fig 2). The remaining 8 mice that did not show a significant decrease in gap detection at 4 weeks post exposure (*p* = 0.26) and were placed in the Tinnitus(-) group.

Next, we assessed how tone exposure affected gap detection within each frequency band for each group. We found no change in gap detection for mice in the Control group over time (F_15,75_ = 1.01, *p* = 0.45; Fig 2A), but a significant time and frequency interaction in Tinnitus(+) mice (F_15,195_ = 2.20, *p* = 0.008; Fig 2B). Follow-up paired t-test showed impaired gap detection at 4 week post-exposure in the following frequency bands: 2–32 kHz (*p* = 0.001), 18–20 kHz (p = 0.02), and 26–28 kHz (p<0.001). By 8 weeks post-exposure, Tinnitus(+) mice remained impaired (p = 0.001) but only for the broadband noise and highest frequency band (2–32 kHz: p = 0.04 and 26–28 kHz: *p* = 0.02). Thus, the onset of tinnitus-like symptoms in mice exposed to a medium frequency tone resulted in broadband and high frequency tinnitus 8 weeks post-exposure.

Mice in the Tinnitus(-) group exhibited selective changes in gap detection (Fig 2C). There was a significant timepoint and frequency interaction in the Tinnitus(-) group (F_15,105_ = 2.64, *p* = 0.002). Gap detection in Tinnitus(-) mice decreased for BBN at 4 and 8 weeks post exposure (*p* = 0.02; *p* = 0.02). Interestingly, gap detection in Tinnitus(-) mice actually improved at 8 weeks post-exposure in the 6-8kHz band (*p* = 0.01). There were no significant gap detection changes for noise in other bands. Unlike Tinnitus(+) mice, Tinnitus(-) mice did not exhibit gap detection impairment for noise in specific frequency bands.

### Tone exposure did not lead to impairments in ABR thresholds

**Fig 3:**
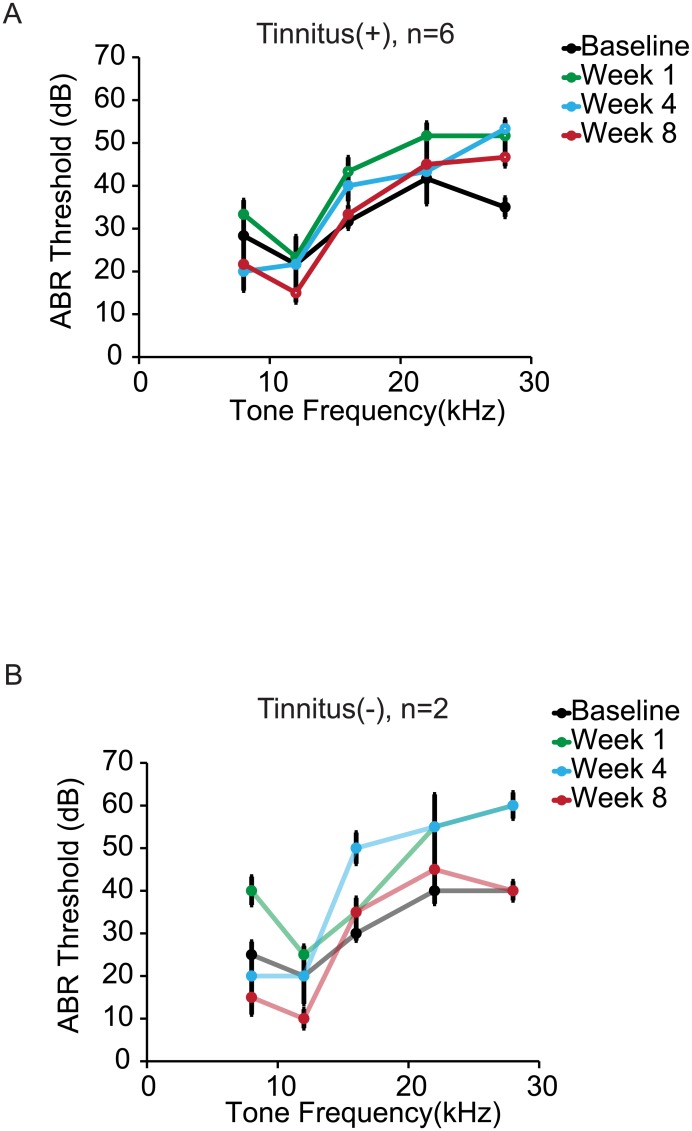
Tone evoked ABR thresholds were measured in a subset of exposed mice at baseline, and at 1, 4, and 8 weeks post-exposure. (A) Tone exposure in Tinnitus(+) mice induced significant hearing loss at 28 kHz at all post-exposure timepoints, and at 16 kHz 1 week post-exposure. No hearing impairments developed for frequencies between 8 kHz and 22 kHz at 4 and 8 weeks post-exposure. (B) Tone exposure also induced hearing impairments in Tinnitus(-) mice for 28 kHz tone 1 and 4 weeks post-exposure, but not at 8 weeks post-exposure. (C-D) Frequency discrimination thresholds are shown for the subset of mice used for ABR recordings. Thresholds were defined as the frequency shift that caused 50% inhibition of the maximum ASR. (C) Tone exposure in Tinnitus(+) mice led to a decrease in frequency discrimination for the 12 kHz tone at 8 weeks post-exposure, and at 4 weeks post-exposure for the 22 kHz tone. (D) Frequency discrimination thresholds did not significantly change after tone exposure in Tinnitus(-) mice. Each data point represents population mean ± SEM. Open circles represent a significant difference from baseline and closed circles a non-significant difference from baseline (significance at p<0.05); *n* refers to the number of mice; *: p<0.05.

Next, we measured ABR thresholds in a subset of mice in the Tinnitus(+) and Tinnitus(-) groups (n = 6; n = 2, respectively) to assess potential hearing loss following tone exposure (Fig 3). We found evidence for sustained elevation in ABR thresholds only for tones at the highest frequency (28 kHz: Week 1 post-exposure: *p* = 0.01; Week 4 post-exposure: *p* = 0.01; Week 8 post-exposure: *p* = 0.01), but not for tones between 8 and 22 kHz (Fig 3A). ABR threshold decreased for tones at 12 kHz at 8 weeks post-exposure when compared to baseline (*p* = 0.02). There was an increase in ABR threshold at 16 kHz at 1 week post-exposure (p = 0.03), but returned to baseline at 4 (*p* = 0.09) and 8 (*p* = 0.36) weeks post-exposure. These results suggest that hearing was not impaired after tone exposure in Tinnitus(+) mice at frequencies between 8 and 22 kHz.

### Selective impairments in frequency discrimination following tinnitus induction

To assay frequency discrimination behaviorally, we modified the ASR measurement to test the mice’s ability to detect a shift in frequency between pre-pulse stimuli and a background tone. Detection of a pre-pulse sound, presented just before a loud startle sound, results in inhibition of ASR. We therefore used ASR inhibition to determine discriminability between the frequency of the background tone and the pre-pulse tone, as the frequency of the pre-pulse tone was varied. Two background frequencies were used: a 12 kHz tone to characterize discrimination for a frequency that falls near the band of exposure, and a 22 kHz tone to characterize discrimination at a frequency higher than that used for tone exposure (where mice exhibited evidence for tinnitus). A repeated measures ANOVA was conducted to evaluate the effects of post-exposure time on PPI.

### Lack of sustained impairments in frequency shift detection in medium frequency bands

**Fig 4:**
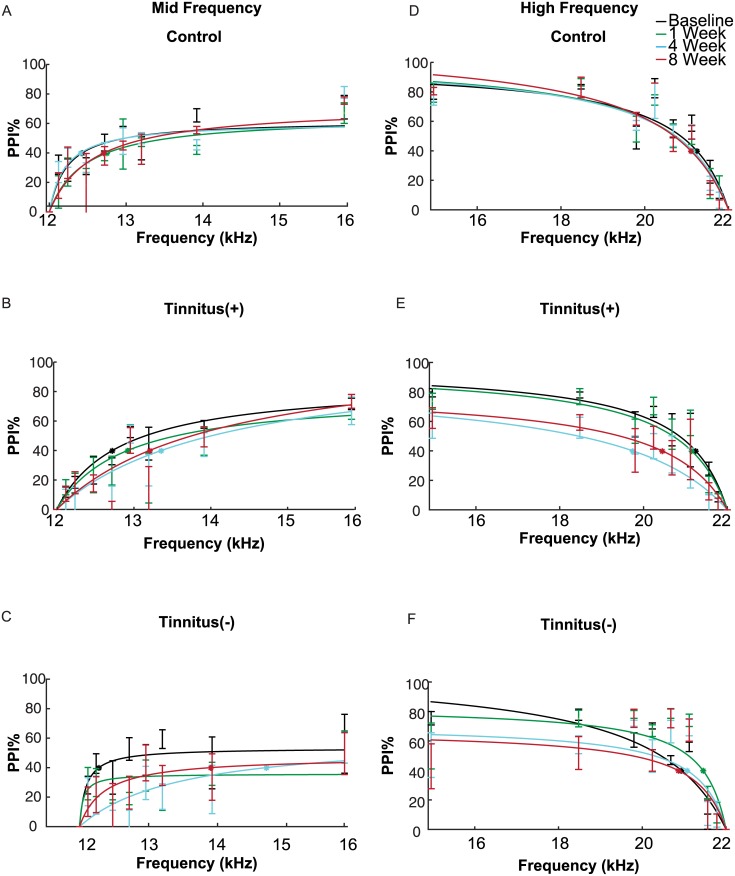
Average pre-pulse inhibition (PPI) of the acoustic-startle response to pre-pulse frequency shifts. (A-C) PPI due to increasing frequency shifts from a 12 kHz background tone for Control (N = 6), Tinnitus(+) (N = 14), and Tinnitus(-) (N = 8) groups at baseline and at 1, 4, and 8 weeks post-exposure. (A) Frequency shift detection remained unchanged in Control mice. (B) Tone exposure in Tinnitus(+) mice led to impaired frequency shift detection at 4 weeks post-exposure, but not at 8 weeks post-exposure. (C) In Tinntius(-) mice, tone exposure also led to a significant decrease in frequency shift detection at 4 weeks post-exposure, but not at 8 weeks post-exposure. (D-F) PPI due to decreasing frequency shifts from a 22 kHz background tone at baseline and at 1, 4, and 8 weeks post-exposure. (D) Frequency shift detection did not change over time in Control mice. (E) Tinnitus(+) demonstrated sustained impairments in frequency shift detection at 4 weeks and 8 weeks post-exposure. (F) There was no significant difference in post-exposure frequency shift detection relative to baseline in Tinnitus(-) mice. Each data point represents population mean ± SEM. *: denotes frequency discrimination thresholds

**Fig 5:**
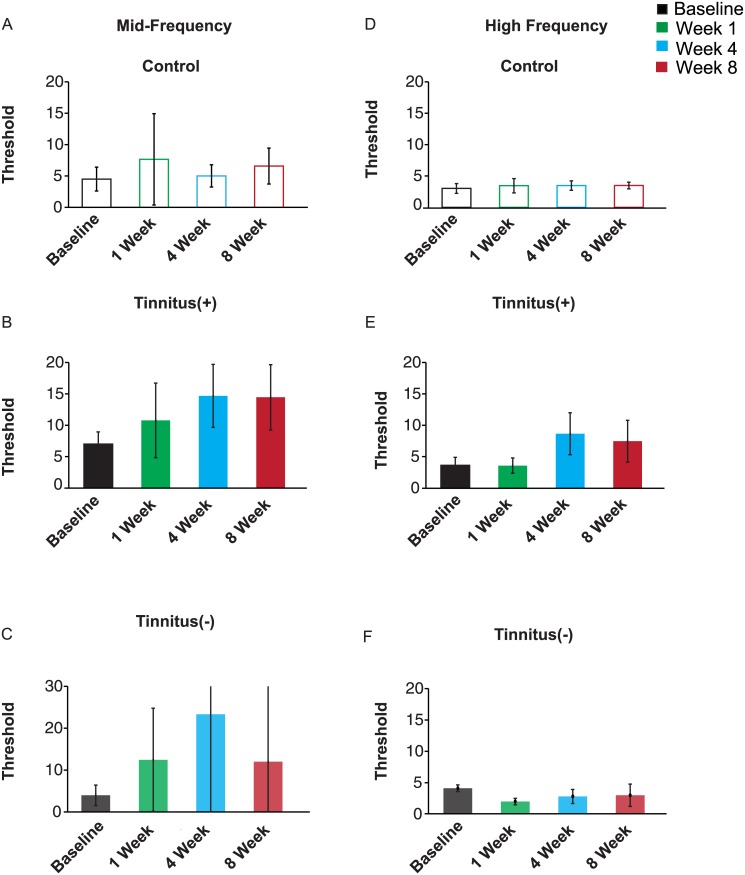
Frequency discrimination threshold, in % frequency change (*Th*_*40*_). Data from panels A, B, C: Frequency discrimination in mid-frequency range. D, E, F: Frequency discrimination in high-frequency range. A, D: Control group. B, E: Tinnitus(+) group. C, F: Tinnitus(-) group. Error bars: standard deviation taken from 1000 repeats generated using parametric bootstrap method.

In mice that exhibited behavioral signs of tinnitus, frequency shift detection around the 12 kHz background tone was impaired following tinnitus induction, but the impairment was not significant at 8 weeks post-exposure (Fig 4B). In the Tinnitus(+) group, there was a significant effect of post-exposure time on PPI (repeated measure ANOVA, time as factor, F_2.72,226_ = 4.31, *p* = 0.01). PPI was significantly reduced at 4 weeks post-exposure (*p* = 0.01), but not at 1 week post-exposure (*p*>0.99) or 8 weeks post-exposure (*p* = 0.22). At 4 weeks post-exposure, this effect was also weak: pairwise comparison of PPI in each pre-pulse frequency at 4 weeks post-exposure were overall not significantly lower than baseline (12.12 kHz: *p* = 0.42; 12.24 kHz: *p* = 0.13; 12.48 kHz: *p* = 0.07; 12.72 kHz, *p* = 0.09; 12.96 kHz: *p* = 0.71; 13.20 kHz: *p* = 0.11; 13.84 kHz: *p* = 0.11; 15.84 kHz: *p* = 0.49). Mice in the Control group did not exhibit significant changes in frequency discrimination after tone exposure (repeated measure ANOVA, time as factor, F_2.3,80.5_ = 2.20, *p* = 0.11; Fig 4A). The threshold for frequency shift detection at 40% PPI was increased at 4 and 8 weeks post tone exposure, but not in Controls (Fig 5A and 5B, difference from baseline by at least one standard deviation estimated using parametric bootstrap method). These results demonstrate that frequency discrimination at medium frequencies exhibited a weak decrease after tone exposure in mice that exhibited tinnitus.

In Tinnitus(-) mice, frequency shift detection around the 12 kHz tone was impaired at 4 weeks post-exposure but this effect was not significant at 8 week post-exposure (Fig 4C). In the Tinnitus(-) group, there was a significant effect of post-exposure time on PPI (F_3,141_ = 5.23, *p* = 0.01). PPI was reduced at 4 weeks post-exposure (*p* = 0.01). At 8 weeks, Tinnitus(-) mice still exhibited impairments in frequency shift detection, but they were not significant (*p* = 0.06) When comparing performance at 4 weeks post-exposure to baseline for each pre-pulse frequency, Tinnitus(-) mice exhibited decreased PPI for the 12.72kHz, 13.20 kHz, and 13.84 kHz pre-pulse tones (*p* = 0.03; *p* = 0.04; *p* = 0.01, respectively) but not at other frequencies (12.12 kHz: *p* = 0.67; 12.24 kHz: *p* = 0.12; 12.48 kHz: *p* = 0.06; 12.96 kHz: *p* = 0.18; 15.84 kHz: *p* = 0.89). The threshold for frequency discrimination at 40% PPI was elevated post-tone exposure, but exhibited high variability (Fig 5C, difference from baseline less than one standard deviation estimated using parametric bootstrap method). These findings suggest that prolonged tone exposure results in temporary deficits in frequency discrimination for frequencies closest to the one used for exposure irrespective of tinnitus induction.

### Sustained impairments in frequency shift detection in high frequency bands due to tinnitus

In the high frequency band (22 kHz), tinnitus induction resulted in sustained impairments in frequency shift detection (Fig 4E). A significant effect of post-exposure time on frequency shift detection was found for Tinnitus(+) mice (repeated measure ANOVA, time as factor, F_2.57,213_ = 23.7, *p*<0.001), but not for the Control group (F_2.41,84.3_ = 0.29, *p* = 0.83; Fig 4D). In Tinnitus (+) mice, PPI was reduced at 4 weeks post-exposure (*p*<0.001) for the following pre-pulse frequencies: 21.78 kHz: *p* = 0.02; 21.56 kHz: *p* = 0.01; 20.24 kHz: *p* = 0.01; 18.48 kHz: *p* = 0.02; 14.96 kHz: *p* = 0.02; and at 8 weeks post-exposure at 21.56 kHz: *p* = 0.02; 20.24 kHz: *p* = 0.03; 18.48 kHz: *p* = 0.01; 14.96 kHz: *p* = 0.02. The frequency shift detection thresholds were elevated in Tinnitus(+) mice at 4 and 8 weeks post-exposure, but not in Controls (Fig 5D and 5E, difference from baseline by at least one standard deviation estimated using parametric bootstrap method). These results demonstrate frequency shift detection at high frequencies remained impaired in Tinnitus (+) mice.

Tone exposure affected frequency shift detection at high frequencies in Tinnitus(-) mice (F_2.5,119.63_ = 4.36, *p* = 0.02; Fig 4F), but did not lead to impairments. When compared to baseline, frequency shift detection in Tinnitus(-) mice was not significantly different following tone exposure (1 week post exposure: *p* = 0.06; 4 weeks post-exposure: *p* = 1.00; 8 weeks post-exposure: *p* = 1.00). Instead, the interaction effect was due to a difference in frequency shift detection at 1 week post-exposure relative to 4 and 8 weeks post-exposure. Detection at 1 week post-exposure was higher than at 4 weeks post-exposure (*p* = 0.05) for the 21.56 kHz pre-pulse frequency (*p* = 0.01) but not for any other pre-pulse frequencies (21.78 kHz: *p* = 0.94; 21.12 kHz: *p* = 0.28; 20.68 kHz: *p* = 0.19; 20.24 kHz: *p* = 0.29; 19.80 kHz: *p* = 0.23; 18.48 kHz: *p* = 0.08; 14.96 kHz: *p* = 0.17). Frequency shift detection at 1 week post-exposure was also significantly higher than at 8 weeks but no significant differences in PPI were found when comparing pre-pulse frequencies (21.78 kHz: *p* = 0.43; 21.56 kHz: *p* = 0.15; 21.12 kHz: *p* = 0.27; 20.68 kHz: *p* = 0.43; 20.24 kHz: *p* = 0.14; 19.80 kHz: *p* = 0.79; 18.48 kHz: *p* = 0.12; 14.96 kHz: *p* = 0.14. The frequency shift detection threshold did not change at 4 or 8 weeks post-exposure in Tinnitus (-) mice (Fig 5F, difference from baseline less than one standard deviation estimated using parametric bootstrap method). These findings demonstrate that in Tinnitus (-), tone exposure did not lead to sustained impairments in frequency shift detection at high frequencies.

## Discussion

Acoustic trauma caused by exposure to loud sounds can cause tinnitus [18, 21, 25, 26] that can be accompanied by hearing deficits. The present study was designed to characterize the effect of tone-induced tinnitus on frequency discrimination acuity in the mouse. We induced tinnitus by prolonged exposure to a loud 10 kHz tone. Consistent with previous studies, mice exhibited selective deficits in gap detection noise bands, which were taken as behavioral evidence of tinnitus [13, 15]. The gap detection deficits were evident in the BBN (2–32 kHz) and the high frequency bands (18–28 kHz) but not in frequency bands below 18 kHz (Fig 2). We then measured changes in frequency discrimination acuity in two frequency bands: in the frequency band close to the tone exposure frequency (12 kHz) and in the high frequency band (22 kHz)—at which the mice exhibit selective deficits in gap detection. Our results indicate that tinnitus induction at high frequencies adversely affects frequency discrimination acuity.

Frequency discrimination impairments were more persistent around the presumed tinnitus (high frequency band) than that of the tone exposure frequency (medium frequency band) (Figs 4 and 5). Discriminability of tones around the high frequency tone (22 kHz) was impaired at 4 and 8 weeks post-exposure. The observed deficits in Tinnitus(+) mice cannot be attributed to hearing loss, since ABR thresholds at 22 kHz and lower frequencies did not change relative to baseline at 4 and 8 weeks post-exposure (Fig 3). Instead, this depression in discriminability is more likely a consequence of tinnitus-like symptoms in the high frequency bands. Evidence for this is further supported by the relatively stable frequency discrimination of Tinnitus(-) mice who, like the Tinnitus(+), underwent tone exposure but did not exhibit behavioral evidence of tinnitus at high frequencies.

By contrast, frequency discrimination at 12 kHz was mildly impaired in Tinnitus(+) mice initially, following noise exposure. Significant deficits in discrimination were apparent at 4 weeks but not 8 weeks post-exposure. These results were also true for the Tinnitus(-) group, suggesting that this effect is probably due to acoustic trauma. Prolonged tone exposure drives various acute and chronic neural changes along multiple levels of the auditory pathway—changes that are likely to trigger and maintain tinnitus symptoms. These changes include spontaneous hyperactivity in brainstem structures such as the inferior colliculous [27], increased synchrony in neural firing rate in the auditory cortex [10, 21], and tonotopic reorganization in the auditory cortex [28–30]. Furthermore, these modulations in neural activity are sustained despite ablation of the dorsal cochlear nucleus [31].

Previous studies have shown that hearing loss modifies the tuning properties of neurons in such a way that they become responsive to frequencies of their unaffected neighboring neurons [21, 30]. In this study, preservation of frequency shift detection around 12 kHz in tone-exposed mice may be a behavioral consequence of neural reorganization in the auditory cortex due to tinnitus. This may also explain the significant improvement in gap detection in Tinnitus(-) mice at 8 weeks—which is behavioral evidence of hyperacusis [32, 33]. The reduction in frequency discrimination acuity around 12 kHz tone is likely due to prolonged tone exposure rather than tinnitus.

The lack of observed evidence for tinnitus in the 10–12 kHz band was surprising—although there was a slight decrease at 4 weeks post-exposure. In a previous study, rats exposed to a 10 kHz tone developed gap detection deficits at high frequencies as well as in the tone exposure frequency band [14]. This could be due to a methodological difference in tinnitus induction; unlike the aforementioned study, the mice used here were not anesthetized during tone exposure but instead were freely moving. Another interesting observation was that gap detection for the BBN background tone was also impaired across all tone exposed mice at 4 and 8 weeks post-exposure.

Tinnitus is a complex disorder manifested via heterogeneous symptoms that are likely due to neural changes within the central auditory pathway [6, 10, 34]. The use of animal models of tinnitus has been vital in unraveling some of these changes. Our study contributes to this growing body of work by suggesting that the tinnitus percept selectively affects frequency discrimination by driving selective hearing impairments.

## Supporting Information

S1 Dataset Sheet 1: data for gap detection, used for Fig 2. Sheet 2: data for pre-pulse inhibition on frequency discrimination task, used for Figs 4 and 5. Sheet 3: data for ABR thresholds, used for Fig 3.
(XLSX)
