# Correction: Selective Impairment in Frequency Discrimination in a Mouse Model of Tinnitus

**DOI:** 10.1371/journal.pone.0139499

**Published:** 2015-09-25

**Authors:** Laetitia Mwilambwe-Tshilobo, Andrew J. O. Davis, Mark Aizenberg, Maria N. Geffen

In the Funding section, the grant number from the NIH is listed incorrectly. The correct grant number is: R01DC014479.

## References

[1] Mwilambwe-Tshilobo L, Davis AJO, Aizenberg M, Geffen MN (2015) Selective Impairment in Frequency Discrimination in a Mouse Model of Tinnitus. PLoS ONE 10(9): e0137749 doi: 10.1371/journal.pone.0137749 26352864 10.1371/journal.pone.0137749 PMC4564173

